# Recognition, rewards, and job performance: the mediating role of perceived organizational support in the Peruvian education sector

**DOI:** 10.3389/fpsyg.2026.1884038

**Published:** 2026-08-24

**Authors:** Pastor Alave-Jimenez, Dany Yudet Millones-Liza, Elizabeth Emperatriz García-Salirrosas, Miluska Villar-Guevara

**Affiliations:** 1 Unidad de Ciencias Empresariales, Escuela de Posgrado, Universidad Peruana Unión, Lima, Peru; 2 Escuela Profesional de Administración, Facultad de Ciencias Empresariales, Universidad Peruana Unión, Lima, Peru; 3 Faculty of Management Science, Universidad Autónoma del Perú, Lima, Peru; 4 Escuela Profesional de Administración, Facultad de Ciencias Empresariales, Universidad Peruana Unión, Juliaca, Peru

**Keywords:** employees, job performance, perceived organizational support, Peru, recognition, reward

## Abstract

**Introduction:**

Publicly recognizing employees’ achievements and contributions—whether through awards, praise, positive remarks at public meetings, or simply words of thanksâ•flmakes them feel valued and appreciated. This not only improves their performance but also strengthens their sense of belonging to the organization and their commitment to its goals and values. Therefore, the objective of this study was to determine the impact of recognition and rewards on job performance: the mediating role of perceived support from a Peruvian educational organization.

**Methods:**

This study employed a quantitative, explanatory-cross-sectional design. It analyzed the responses of 266 employees belonging to an educational association in Peru. The theoretical model was evaluated using the Partial Least Squares method (PLS-SEM).

**Results:**

A measurement model with adequate fit was obtained (*α* = between 0.886 and 0.950; CR = between 0.903 and 0.951; AVE = between 0.768 and 0.813), and six of the seven study hypotheses were supported by the structural model, with the direct effect of rewards on employee performance (H2) not being statistically significant.

**Discussion:**

In this regard, the study provides valuable information for Peruvian educational leaders seeking to improve indicators of job performance and perceived organizational support. These findings enrich educational management, given that, through this integrated view of the model, it will be possible for educational organizations to achieve sustainable levels of high job performance, which, in turn, will result in the well-being and development of employees.

## 1 Introduction

Today, the success or failure of an organization depends largely on various factors involving employees, and in many cases, perceived support plays a predominant role (Melton, 2017; Gomes et al., 2021; Li et al., 2022). Perceived support, refers to employees’ sense that the organization supports them, cares about them, and works for their well-being (Choi, 2020; Rasouli et al., 2020; Chen and Eyoun, 2021). When employees feel supported by their managers, supervisors, immediate supervisors, and the company, they are more motivated, committed, and willing to give their best at work (Liu et al., 2023; Solís et al., 2023; Huang et al., 2023). This support may take the form of providing resources, offering constructive feedback, allowing for flexible work schedules, and ensuring a healthy work-life balance (Al Doghan, 2019; Caesens et al., 2019; Oubibi et al., 2022; Sarwar et al., 2023).

On the other hand, recognition is an effective way to reinforce desired behavior and motivate employees to maintain their performance (Magalhães-Alves et al., 2017; Seabrooke et al., 2019). Publicly acknowledging employees’ achievements and contributions—whether through awards, praise, positive remarks at public meetings, or simply words of thanks—makes them feel valued and appreciated (Pinheiro, 2020; Delavallade, 2021). This not only improves their performance (Wu et al., 2021), but also strengthens their sense of belonging to the organization and their commitment to its goals and values (Nouri and Parker, 1998; Putri and Suartana, 2018; Astuty and Udin, 2020; Bonhak et al., 2020).

Recent studies highlight the importance of rewards, which can take various forms, including salary increases, bonuses, promotions, overtime, professional development, etc. (Santa Maria et al., 2021; Elrayah and Semlali, 2023; Hambali et al., 2023). These rewards are important for recognizing high-performing employees and fostering continuous improvement (De Clercq et al., 2023). By linking good performance to good rewards, organizations create an incentive system that encourages employees to work toward specific goals and contribute to the success of the organization or business (Saether, 2020). Recognizing employees’ achievements, whether significant or not, can be truly rewarding for them; and these rewards help employees stay motivated and perform better (Manzoor et al., 2021; Peng et al., 2023). The reviewed literature shows a consistent relationship between employee performance and recognition (Clarke et al., 2019; Clarke and Mahadi, 2017; Jin and Kim, 2022; Wihler et al., 2014) and rewards (Chi et al., 2023; Feuerhahn et al., 2012; Bonhak et al., 2020).

In this regard, previous research on these topics considers perceived support, recognition, and rewards as important factors for improving employee performance and engagement. By creating a work environment where employees feel supported, valued, and rewarded for their efforts and contributions, organizations can promote motivation, job satisfaction (Muguerza-Florián et al., 2025b), and high performance, which leads to business success (Chi et al., 2023; Santa Maria et al., 2021; Sureephong and Dahlan, 2020). Based on this scenario, in educational organizations, effective human talent management has become an essential component for strengthening teachers’ job performance and promoting institutional effectiveness (Agustin-Silvestre et al., 2024; Laura-Arias et al., 2024; Alimbekov et al., 2025). Within this framework, recognition and rewards constitute organizational practices that communicate appreciation for employees’ contributions, while perceived organizational support reflects teachers’ beliefs about the degree to which their institution values their work and cares about their well-being (Zhang et al., 2023). Previous research has shown that perceived organizational support is positively associated with employee attitudes and performance, while recognition practices contribute to reinforcing employees’ perception of organizational appreciation (Yang et al., 2022; Li et al., 2025). In the Peruvian educational context, characterized by diverse organizational realities and increasing demands on teachers, examining the interaction between recognition, rewards, perceived organizational support, and job performance can contribute to a better understanding of the organizational factors associated with teacher performance within educational institutions (Li et al., 2025).

Although the relationships among recognition, rewards, perceived organizational support, and job performance have been extensively examined in corporate and industrial settings, empirical evidence from the Peruvian educational sector remains limited, particularly within Regular Basic Education institutions. This gap is especially relevant because educational organizations differ from business environments in their institutional mission, value-based orientation, service focus, and the central role of teachers in achieving educational outcomes. These organizational characteristics suggest that employees’ responses to recognition, rewards, and perceived organizational support may not necessarily mirror those reported in profit-oriented organizations. Furthermore, despite the growing application of structural equation modeling in organizational research, few studies have examined these relationships simultaneously using a PLS-SEM framework in the Peruvian educational context. Accordingly, this study addresses an important empirical gap by testing an integrated structural model that examines the direct and mediating influences among these constructs within an educational organization, thereby extending current knowledge on human resource management in educational settings. In this regard, the purpose of the research was to determine the impact of recognition and rewards on job performance: The mediating role of perceived support from educational organizations.

## 2 Theoretical framework and hypothesis development

Compensation systems are a series of actions that organizations provide to their employees, which are perceived as a highly valued sign of support; thus, rewards are tangible benefits that improve employees’ perception of organizational support (Jianmin, 2024). In this regard, financial or non-financial rewards (days off, gift cards, promotions) that are valued by employees can increase the perception of organizational support, as in this scenario, employees feel that their contributions are valued and that the organization cares about their well-being (Hoa et al., 2020; Jabagi et al., 2020). Indeed, when a company develops effective reward systems, it leads employees to maintain a stronger emotional attachment, which reinforces their perception of support (Ndiango et al., 2024; De la Torre-Ruiz et al., 2025). And when employees strengthen their attachment, there is a high probability that they will identify with the organization’s values and goals and commit to it, thereby striving to perform better work (Yang and Jiang, 2023).

Furthermore, the existing literature suggests that employees who receive rewards feel valued and are less likely to experience burnout, while also reducing their intention to leave (Wei et al., 2025), as it has been demonstrated that rewards satisfy material needs and have a significant effect on physical well-being; when a worker feels rewarded, they strive much harder to achieve efficiency (De Angelis et al., 2021). On the other hand, previous studies establish that rewards satisfy a material need, which enhances intrinsic motivation and improves job performance, as motivation drives workers’ determination (Ce et al., 2025). Additionally, it is important that rewards be distributed equitably, as it has been demonstrated that a worker who perceives a fair reward system will perform better compared to a worker whose bonuses or rewards are eliminated (Bareket-Bojmel et al., 2017). However, all these behaviors depend on the context and the individuality of the workers, and it has been shown in different contexts that rewards do not always have a direct impact on performance, but can influence it indirectly through motivation and commitment (Ul Haq et al., 2023; Aklima et al., 2026).

Based on the above, the following hypotheses are proposed:

*H1:* Employee rewards influence perceived organizational support.

*H2:* Rewards influence employee performance.

Recognition is an intangible asset centered on emotional and social value; for example, a congratulatory email, a certificate of recognition, or another form of acknowledgment that recognizes an employee’s effort will generate intrinsic motivation that enhances the employee’s performance (Chan and Hooi, 2023; Black et al., 2024). It has been demonstrated that sustained motivation improves sustained performance among employees and strengthens organizational culture (Bocean et al., 2021; Zeng, 2021). Although recognition has been shown to be central to motivation, which varies according to the worker’s personal needs and desires, all motivation has a specific positive effect: improving worker and team performance, leading to a significant improvement in work dynamics and, consequently, higher levels of performance and innovation (Jasińska, 2019).

Furthermore, the scientific literature indicates that recognition is a vital component of team success, as it serves as a foundation for establishing clear communication patterns that facilitate conflict resolution and improve team dynamics (Rajan et al., 2025). Furthermore, recognition acts as a “cultural glue” that unites organizational members to achieve institutional goals, leading to improved employee performance (Schaetzle, 2016; Qu et al., 2025). In turn, recognition promotes and intensifies employees’ perception of support from the organization where they work, as they feel the organization’s concern for them, which fosters a sense of belonging (Ndiango et al., 2024). Based on the above, the following hypotheses are proposed:

*H3:* Recognition influences employee performance.

*H4:* Recognition influences perceived organizational support.

Perceived organizational support has a direct influence on job performance; when an employee perceives organizational support, they tend to perform their duties better (Shore et al., 2025), feel a greater sense of responsibility, and strive to fulfill all their work obligations (Goyal et al., 2023). Other studies indicate that perceived support also has moderating and mediating effects; that is, it acts as a mediator in various relationships, for example, between reward and performance, highlighting its role in achieving greater effectiveness (Marique et al., 2013; Nazir and Islam, 2017; Hakim, 2025). Therefore, when an organization makes efforts to improve employees’ perception of support, it ensures, to a certain extent, significant improvements in employee performance (Vatankhah et al., 2017; Eisenberger et al., 2025); which means that when perceived social support is activated, rewards and recognition positively contribute to achieving better employee performance. Given these statements, the following hypotheses are proposed:

*H5:* Perceived organizational support influences employee performance.

*H6:* Perceived organizational support mediates the relationship between rewards and employee performance.

*H7:* Perceived organizational support mediates the relationship between recognition and employee performance.

## 3 Materials and methods

### 3.1 Study design

This study employed a quantitative, explanatory cross-sectional design (Ato et al., 2013), with statistical analysis conducted using the partial least squares structural equation modeling (PLS-SEM) approach. The evaluation was conducted in two stages: assessment of the psychometric properties of the measurement scales to identify reliability, convergent validity, and discriminant validity; followed by hypothesis testing using structural equation modeling (SEM).

### 3.2 Process and ethical considerations

This study was reviewed and approved by the Ethics Committee of a private university in Peru (Certificate 2023-CE-EPG-00041) to ensure that the study did not expose the population to any risk. Prior to data collection, written authorization was requested from the senior management of each participating institution to support the distribution of the online questionnaire. This research also ensured compliance with the principles of the Declaration of Helsinki, preserving the anonymity of participating workers and adhering to confidentiality standards. Accordingly, each worker who voluntarily participated was fully informed about the research objective and provided informed consent. In this regard, between May and July 2024, participants were invited to complete the online questionnaire.

### 3.3 Study sample

This study included Peruvian employees belonging to an educational association. This is a nationwide network that shares common characteristics, including providing values-based education. Additionally, other inclusion criteria were being over 18 years of age, maintaining some type of connection with the institution, and having either a fixed-term contract (staff with a fixed-term contract) or being a permanent employee (staff who, due to their years of service, hold a permanent contract). For data collection, a non-probabilistic convenience sampling method was selected (Otzen and Manterola, 2017). The study was conducted within the Lima Sur Adventist Educational Network, a network comprising a total of 8 schools and 350 employees, from which the sample was selected based on the following inclusion criteria: staff with a valid contract at the time of the survey, Peruvian employees, and those who provided informed consent. Exclusion criteria included employees on leave, foreign employees, and those who did not provide informed consent. Participants were recruited with the support of the school principals, who were responsible for sharing the link to the online questionnaire through institutional WhatsApp groups. The questionnaire was self-administered and hosted on the Google Forms platform, with all items set as required, thus ensuring that no incomplete or invalid responses were recorded. Data were collected over a three-month period (May–July 2024), with 266 workers participating; all of their responses were valid and included in the analysis, representing a response rate of 76.0% (266/350).

As shown in Table 1, participation among women (53.76%) and men (46.24%) was largely equal. However, the highest participation was concentrated among employees aged 31 to 40 (31.58%), who were married (54.14%), and who had fixed-term contracts (72.56%).

**Table 1:** Demographic characteristics of the 266 participants.

Characteristic	Frequency	Percentage (%)
Gender		
Female	143	53.76
Male	123	46.24
Age		
21–30	78	29.32
31–40	84	31.58
41–50	78	29.32
51- and up	26	9.77
Marital status		
Single	103	38.72
Married	144	54.14
Widowed	4	1.50
Divorced	6	2.26
Living with a partner	9	3.38
Employment status		
Hired	193	72.56
Employee	73	27.44

### 3.4 Measurement scales

The instruments were extracted from an English version and therefore underwent a back-translation process by a professional with no connection to this study; following this, an online focus group session was conducted with six representative teachers who met the inclusion criteria, ensuring semantic validity and a clear understanding of each item. Finally, an online questionnaire was designed, with the data hosted on Google Forms. This questionnaire was shared via WhatsApp, Telegram, and Messenger, thereby facilitating the sample group’s participation. This online questionnaire was divided into three sections: The first section described the informed consent process, where each participant acknowledged the purpose of the study and authorized the processing of the information they would provide; the second section contained the items for each metric (see Appendix A); and finally, the third section requested sociodemographic information from the participants.

A Likert-type response scale was used to measure each item, where 1 represented “strongly disagree” and 5 represented “strongly agree.” Four items were used to assess recognition; four items for perceived organizational support; seven items for rewards; and three items for employee performance. The instruments were adapted from Hussain et al. (2019)and achieved optimal internal consistency indicators, which were measured using Cronbach’s alpha (*α* = between 0.886 and 0.950).

### 3.5 Statistical analysis

This study employed Partial Least Squares Structural Equation Modeling (PLS-SEM) for data analysis. PLS-SEM is a variance-based structural equation modeling approach that focuses on maximizing the variance explained by endogenous constructs (Henseler et al., 2015). Its main benefits include: (1) its predictive approach, which prioritizes explaining variance in dependent variables (Shmueli et al., 2019); (2) its versatility in simultaneously handling formative and reflective measurement models; and (3) its effectiveness in managing complex models involving various mediating relationships (Sarstedt et al., 2022). The PLS-SEM analysis was conducted in two stages, following best practices (Hair et al., 2019). First, the psychometric properties of the measurement instrument were examined, including the assessment of reliability, convergent validity, and discriminant validity of the constructs. Finally, the hypotheses formulated in the study were tested using structural equation modeling.

## 4 Results

### 4.1 Model evaluation

According to Hair et al. (2017), to evaluate the measurement model, an estimation of construct reliability (composite reliability and Cronbach’s alpha) and validity (discriminant and convergent validity) was proposed. The Cronbach’s alpha (α) values range from 0.886 to 0.950, and the threshold value of 0.70 falls below these values (Hair et al., 2017). Likewise, the composite reliability (CR) yields values between 0.903 and 0.951, which were above the suggested value of 0.70 (Kline, 2015). Based on the aforementioned indicators, all constructs were considered error-free, thus establishing the construct’s reliability (see Table 2).

**Table 2:** Convergent validity results.

Construct	Items	Factor loading	α	CR	AVE
Employee performance (EP)	EP1	0.910	0.886	0.903	0.813
	EP2	0.886			
	EP3	0.909			
Perceived organizational support (POS)	POS1	0.874	0.913	0.913	0.793
	POS2	0.873			
	POS3	0.911			
	POS4	0.903			
Recognition (RC)	RC1	0.881	0.916	0.919	0.800
	RC2	0.886			
	RC3	0.874			
	RC4	0.935			
Rewards (RW)	RW1	0.880	0.950	0.951	0.768
	RW2	0.908			
	RW3	0.890			
	RW4	0.840			
	RW5	0.873			
	RW6	0.881			
	RW7	0.860			

The convergent validity results ensured acceptable values (factor loading, Cronbach’s alpha, and composite reliability (CR) ≥ 0.70 and average variance extracted (AVE) > 0.5).

Average Variance Extracted (AVE) and factor loadings must be tested for convergent validity (Hair et al., 2017). According to Table 2, all factor loadings were above the suggested value of 0.7. Furthermore, Table 2 shows that AVE values range from 0.768 to 0.813 and are above the threshold value of 0.50. The indicators found demonstrate adequate convergent validity for the constructs.

### 4.2 Discriminant validity

To determine this, the Fornell-Larker was considered (Hair et al., 2017). Table 3 shows the requirements according to the conditions established by Fornell-Larcker; the table indicates that all AVEs and their square roots are greater than their correlations with other constructs (Fornell and Larcker, 1981).

**Table 3:** Fornell-Larker scale.

Construct	EP	POS	RC	RW
EP	**0.902**			
POS	0.656	**0.891**		
RC	0.633	0.757	**0.894**	
RW	0.609	0.811	0.855	**0.876**

The values on the diagonal in bold represent the square of the AVE.

### 4.3 Analysis of the structural model

The proposed hypotheses were tested using the PLS-SEM technique. Predictive validity scores were used to fit the model. Cross-validated redundancy values (*R*^2^) represent the model’s predictive validity. *R*^2^ values must be greater than 0 for the model to be accurate (Hair et al., 2014; Henseler et al., 2015). *R*^2^ values were determined using the blindfolding method, where all values of the endogenous construct were greater than 0, indicating the model’s accuracy. Table 4 shows the endogenous latent variables along with their respective R(2) values.

**Table 4:** *R*^2^ of the endogenous latent variables.

Construct	*R* ^2^
Employee performance (EP)	0.474
Perceived organizational support (POS)	0.672

**Figure 1:**
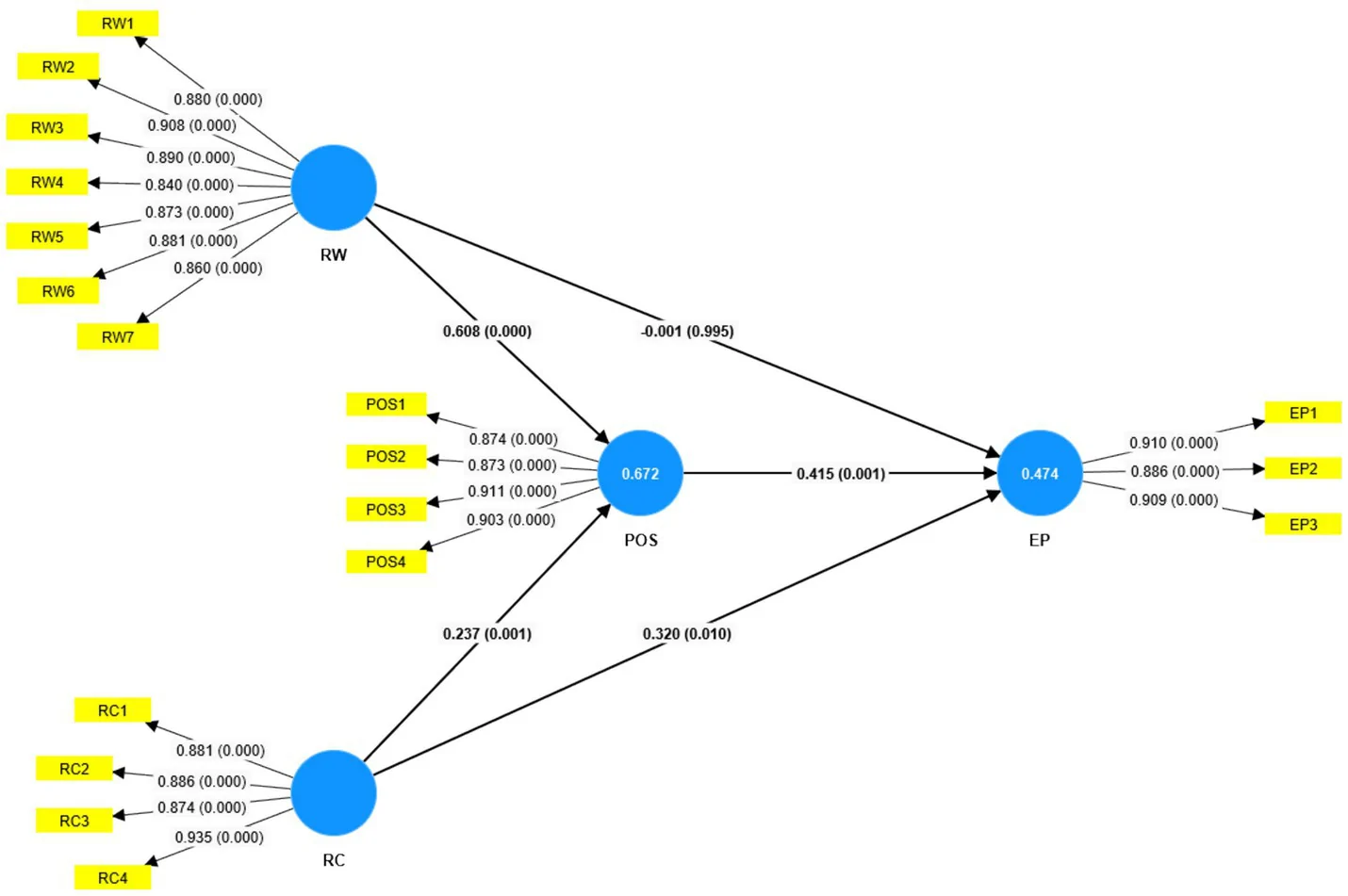
Structural model.

The path coefficient values, *p*-values, and t-statistics were used to accept and reject the hypotheses as shown in Figure 1 and Table 5. The strength of the relationship between the variables can be examined through the path coefficient values. Path coefficient values close to +1 indicate a strong relationship and vice versa (Hair et al., 2016). The *p*-values and t-statistics pertain to the acceptance and rejection of the proposed hypotheses. In this study, the conceptual model contains seven hypotheses. The results of the tested hypotheses are summarized in Table 5. H1 is accepted, which proposed that reward (RW) has a positive impact on perceived organizational support (POS) (*β* = 0.608, *p* = 0.000, *t* = 8.967); H2 is not supported, which proposed that rewards (RW) have a positive impact on employee performance (EP) (*β* = −0.001, *p* = 0.995, *t* = 0.007); H3 is accepted, which proposed that recognition (RC) has a positive impact on employee performance (EP) (*β* = 0.320, *p* = 0.010, *t* = 2.568); H4 is accepted, which proposed that recognition (RC) has a positive impact on perceived organizational support (POS) (*β* = 0.237, *p* = 0.001, *t* = 3.319); H5 is accepted, which proposed that perceived organizational support (POS) has a positive impact on employee performance (EP) (*β* = 0.415, *p* = 0.001, *t* = 3.416); H6 is accepted, which proposed that perceived organizational support (POS) plays a mediating role in the influence of reward (RW) on employee performance (EP) (*β* = 0.252, *p* = 0.001, *t* = 3.209) and H7 is accepted, which proposed that perceived organizational support (POS) plays a mediating role in the influence of recognition (RC) on employee performance (EP) (*β* = 0.098, *p* = 0.007, *t* = 2.712).

**Table 5:** Results of the structural model.

H	Hypothesis	Original sample (O)	Sample mean (M)	Standard deviation (STDEV)	T-statistics (|O/STDEV|)	*p*-values	VIF	Decision
H1	RW → POS	0.608	0.612	0.068	8.967	0.000	3.724	Supported
H2	RW → EP	−0.001	0.015	0.138	0.007	0.995	4.854	Not supported
H3	RC → EP	0.320	0.316	0.125	2.568	0.010	3.896	Supported
H4	RC → POS	0.237	0.233	0.071	3.319	0.001	3.724	Supported
H5	POS → EP	0.415	0.401	0.121	3.416	0.001	3.052	Supported
H6	RW → POS → EP	0.252	0.245	0.079	3.209	0.001	n/a	Supported
H7	RC → POS → EP	0.098	0.091	0.036	2.712	0.007	n/a	Supported

VIF values correspond to the full collinearity test (Kock, 2015), assessed at the level of each structural path (inner model); values below the conservative threshold of 3.3 are considered indicative of a low risk of common method bias. H6 and H7 represent indirect (mediated) effects and were therefore not evaluated individually through this procedure.

Thus, six of the seven hypotheses were confirmed, and these findings highlight the importance of employee rewards and recognition as a key factor in the perception of perceived organizational support, which would have a significant impact on compensation strategy, human resource management, and organizational culture in general. Furthermore, appropriate recognition can act as a powerful motivator for employees, increasing their level of commitment to the organization and their willingness to contribute to the achievement of business objectives. It can also help reduce staff turnover by increasing job satisfaction and employee loyalty. Finally, the perception of strong organizational support can contribute to increased job satisfaction among employees, improve the organizational climate, and boost productivity and performance.

## 5 Discussion

This study examines the associations between recognition and rewards and the performance of 266 regular elementary education teachers, selected from 8 schools affiliated with an educational association. The evaluation was conducted using structural equation modeling (PLS-SEM), which found sufficient evidence to accept 6 of the 7 proposed hypotheses. Because this study relies on a cross-sectional PLS-SEM design, the relationships identified among constructs should be interpreted as statistical associations rather than causal effects; consequently, the discussion below refers to variables being related to or predicting one another in a statistical sense, rather than one variable causing changes in another.

Regarding the predictive validity of the proposed model, the *R*^2^ values reported in Table 4 indicate that the exogenous constructs examined explained 47.4% of the variance in employee performance (*R*^2^ = 0.474) and 67.2% of the variance in perceived organizational support (*R*^2^ = 0.672). These values reflect a moderate contribution of recognition and rewards to explaining employee performance, and a substantial contribution of rewards and recognition to explaining perceived organizational support. At the same time, they indicate that 52.6% of the variance in employee performance and 32.8% of the variance in perceived organizational support remain unexplained by the proposed model, suggesting that other variables not considered in this study—such as job satisfaction, organizational commitment, leadership style, or individual and contextual characteristics of employees—may also contribute to explaining these outcomes and should be incorporated in future research.

The findings provide sufficient evidence of a significant positive association between employee rewards and perceived organizational support (*β* = 0.608, *p* < 0.001), suggesting that when an employee receives a reward, this is accompanied by a more favorable perception of the institution’s support. Because rewards and perceived organizational support were both measured through self-reported perceptions collected from the same respondents at a single point in time, the possible influence of common method bias on this and other relationships in the model was examined empirically. Harman’s single-factor test showed that the first unrotated factor accounted for 64.2% of the total variance among the 18 indicators, exceeding the conventional 50% threshold, and a full collinearity test (Kock, 2015) yielded inner VIF values ranging from 3.052 to 4.854 across the structural paths (see Table 5), with most paths exceeding the conservative threshold of 3.3. Taken together, these results indicate that common method bias cannot be ruled out in the present study; this should be considered alongside the strong theoretical and empirical overlap between rewards and recognition (*r* = 0.855, Table 3), which may also contribute to the elevated collinearity independently of method effects. Given these results, the relatively strong association observed between rewards and perceived organizational support, and the remaining relationships based on self-reported measures, should be interpreted with corresponding caution; in this regard, Saether (2020) states that rewards are used as a motivational tool that leads to a shift in an employee’s perspective toward their institution. To support this assertion, Mabaso and Dlamini (2018) and Chan and Hooi (2023) argue that high rewards lead to motivated employee behavior, which opens the possibility of fostering a more positive perspective among talent toward the organization where they work. This holds true regardless of whether the rewards are monetary, as it is clear that any reward encourages a more favorable perception of the company, thereby promoting a satisfying work environment (Peng et al., 2023).

Furthermore, it has been demonstrated that employee recognition is an opportunity to foster their professional development and performance; through recognition, employees feel valued, thereby increasing the likelihood of perceiving greater support within the organization (Gholizadeh et al., 2022); to support the findings of this study, it has also been found that employees’ perceived support arises as a response to the institution’s appreciation and trust in the employee (Loh and Idris, 2024). Indeed, according to research, recognition is considered a key factor in perceived support; this means that when a person receives recognition for their work, positive feelings and perceptions emerge regarding the organization where they work; thus, it is important for organizational leaders to foster a culture of recognition toward their employees (Slišković et al., 2016).

In contrast, the results of this study indicate that rewards did not significantly predict employee performance (*β* = −0.001, *p* = 0.995), and hypothesis H2 was therefore not supported. This result should not be read as evidence that rewards have no relevance for teachers’ performance, but rather as an absence of a statistically significant direct association between these two variables in the present sample; any interpretation of this finding must remain consistent with this null result. This finding is consistent with Njanja et al. (2013), who similarly found that cash bonuses did not have a significant effect on employee performance, suggesting that rewards alone may not be a decisive driver of performance. Rewards can, however, serve as one component of a broader motivational system that supports performance, as reward systems have been identified as one of several factors influencing job performance in educational settings (Kumari and Kumar, 2023). In this context, it is argued that the association between rewards and employee performance depends on the employees’ perspective and culture and varies according to the organizational context and individual characteristics of the employees (Ul Haq et al., 2023; Aklima et al., 2026).

On the other hand, this study found that recognition was significantly and positively associated with employee performance (*β* = 0.320, *p* = 0.010); according to Clarke and Mahadi (2017), recognition is a symbol of respect and predicts job performance, as a job well done arises in response to the organization’s appreciation and recognition of the employee, which translates into motivation to strive to meet goals (Minsuck and Boyoung, 2022); from this perspective, the authors assert that recognition brings about a significant change in work attitude; in this regard, it is important that an organization’s management take actions to strengthen recognition of employees. Another study supporting this finding is that of Bonhak et al. (2020), who establish that both emotional and material rewards are essential for fostering employee commitment and work performance, as it has been demonstrated that support from a manager or supervisor can promote high performance through better work practices (He et al., 2020; Wu et al., 2021).

Likewise, this study found that, in the study population, perceived organizational support was significantly and positively associated with employee performance (*β* = 0.415, *p* = 0.001); this result is consistent with research indicating that organizational characteristics are related to employee performance; as such, it is specified that positive experiences (Acuña-Hurtado et al., 2024) are a source that fosters positive employee behavior toward the responsibilities they assume (Tremblay and Simard, 2018) and the fact is that employees’ actions regarding their work performance depend to a certain extent on what they perceive and how they feel about it (Lavelle et al., 2007). Thus, it is necessary to take the necessary steps to foster good interpersonal relationships between employees and the company, as it has been demonstrated that perceived support can alleviate employee dissatisfaction, thereby shifting attitudes toward a positive outlook that enables a significant contribution toward institutional goals (Al-Abrrow, 2022).

Regarding perceived organizational support, this study found that it plays a mediating role in the relationship between rewards and employee performance (*β* = 0.252, p = 0.001), indicating that rewards are associated with employee performance indirectly, through employees’ perceptions of organizational support, rather than through a direct statistical association, which is consistent with the non-significant direct path observed for H2. Research supporting this idea shows that rewards are closely related to employee performance; in this regard, to strengthen this connection, it is necessary to address employees’ perception of support within the work; to this end, leaders are responsible for creating a conducive work environment (Liu and Liu, 2022). Furthermore, evidence shows that any reward or incentive is a key determinant of improved work outcomes, which can vary depending on the employee’s perception (Wang et al., 2022). In this context, the need for companies to design reward strategies that are perceived as fair and equitable is emphasized, thereby achieving high job performance (Jaworski et al., 2018).

Finally, the findings also indicate that perceived organizational support plays a mediating role in the relationship between recognition and employee performance (*β* = 0.098, *p* = 0.007); this result can be interpreted in light of motivation theory, which explains that people’s motivation leads them to be more inclined to perform better, thereby achieving greater efficiency through their performance and reducing employee turnover; this link may be altered depending on how they perceive support (Jaworski et al., 2018). In this regard, it has been found that perceived support plays an important role in achieving better performance, which is linked to the recognition they receive; therefore, all such achievements should be recognized, accompanied by constructive feedback (Nguyen et al., 2023), as it has been demonstrated that greater recognition leads to increased work performance, though this effect may be moderated by the capabilities demonstrated by an organization’s managers (Nordgren Selar et al., 2023).

### 5.1 Theoretical, practical, and management implications

Among the implications, several dimensions stand out in which this study contributes to the theoretical and practical fields of human talent management and organizational performance.

From a theoretical perspective, the findings strengthen the literature on organizational behavior by demonstrating that perceived organizational support is a fundamental explanatory mechanism that links recognition practices, rewards, and job performance. In this sense, the study transcends approaches that analyze these constructs in isolation, showing that organizational practices focused on recognition and rewards acquire greater explanatory power when interpreted through the perceptions employees develop regarding the commitment and value the organization provides them. Furthermore, the confirmation of the mediating effects of perceived organizational support contributes to refining conceptual models of job performance by providing empirical evidence that the effects of talent management practices, especially among Peruvian teachers, are not exclusively direct, but also occur through psychological processes that strengthen the bond between the employee and the organization. Taken together, these findings broaden the theoretical understanding of the mechanisms that underpin employee performance and offer a more integrative conceptual framework for future research in organizational behavior and human talent management.

Additionally, the results validate exchange theory by demonstrating that reciprocal relationships between the employee and the organization, mediated by perceived support, generate a virtuous cycle of commitment and performance. Furthermore, the mediating role of perceived support, validated in this study, contributes to the theory of perceived organizational support (POS), in the sense that this construct is not only an independent variable but also acts as an intermediate psychological mechanism that can amplify the effects of recognition and rewards on job performance.

From a practical and managerial perspective, the findings of this research provide very useful recommendations for educational organizations. First, although this study did not find a significant direct effect of rewards on employee performance, managers are still advised to establish and implement integrated recognition and reward systems that incorporate both tangible elements (bonuses, promotions, professional development) and intangible elements [public recognition, certificates, or expressions of gratitude (Muguerza-Florián et al., 2025a)] given their demonstrated influence on perceived organizational support and, indirectly, on performance. Second, the idea that practices in these organizations must be perceived as fair and equitable should prevail. In fact, the perception that such rewards are unfair can have negative effects on performance. Third, organizations must deliberately invest in improving perceptions of organizational support given its mediating effect, which in turn means building a work environment where employees feel that the organization is interested in and cares about their well-being, that their contributions are valued, and that the organization cares about their holistic development.

This study provides educational organizations with concrete evidence linking organizational values that incorporate internal principles of recognition of human worth—especially those stemming from community support—to significant levels of competitive advantage regarding employee performance. The educational institutions that participated in the sample of this study can, based on the results obtained, design human resources policies that allow them to increase their sense of institutional identity and their sense of educational mission by linking recognition and rewards to pedagogical objectives and the organization’s own institutional values.

### 5.2 Limitations and future research

Despite the significant findings, several limitations must be considered for a better interpretation of the results. First, since the study employs a cross-sectional design, it is not possible to assess the temporal stability of the responses from the employees in the educational network who were the subjects of this research. Therefore, future research should consider a longitudinal approach and apply it in different contexts to strengthen its validity and applicability across diverse populations.

Second, given the use of a non-probability sampling method, the findings of this study apply directly to employees in Peru’s education sector, with very specific characteristics inherent to the study population. Therefore, to achieve greater generalizability, additional studies are needed to replicate the results using representative probability samples from Peru.

Third, the study sample consisted predominantly of workers with fixed-term contracts (72.56%), which may have introduced potential response biases associated with social desirability and/or psychological factors linked to past/current experiences in work environments (Acuña-Hurtado et al., 2024), insecurity, fears, dissatisfaction, discomfort, and workplace injustices, among others. These factors may have influenced participants’ perceptions and self-reported responses. Therefore, future research should aim to ensure a more balanced distribution of participants across years of service (contracted and permanent employees) to capture a broader and more representative range of perspectives, thereby improving the generalizability and robustness of the findings.

Finally, given that rewards were not significantly associated with teachers’ job performance in this study, future research should examine in greater depth the factors underlying this relationship, incorporating variables that were outside the scope of this study, such as worldview, personal values, organizational commitment, and teaching vocation, particularly in educational institutions characterized by a distinctive institutional philosophy. Furthermore, qualitative or mixed-methods studies are recommended to explore the organizational dynamics of educational networks and other organizational contexts, providing a deeper understanding of how these contextual and individual factors can interact with reward systems and shape job performance. These approaches could contribute to the development of more comprehensive explanatory models of employee performance in diverse organizational environments.

## 6 Conclusion

This study achieved its objective of examining the relationships among rewards, recognition, perceived organizational support, and employee performance in teachers of an Adventist Educational Network in South Lima. The findings confirmed six of the seven proposed hypotheses; the exception was the hypothesized direct association between rewards and employee performance, which was not statistically significant, highlighting perceived organizational support as a key mechanism linking recognition and rewards with employee performance. Specifically, perceived organizational support was positively associated with employee performance and mediated the relationships between rewards and performance, as well as between recognition and performance, reinforcing its central role within the proposed theoretical model.

Overall, these findings contribute to the literature on human resource management by providing empirical evidence on the interplay among recognition, rewards, perceived organizational support, and employee performance in a Latin American educational context. Rather than acting solely as direct organizational practices, recognition and rewards appear to be most strongly associated with employee performance when accompanied by employees’ perceptions of organizational support, thereby advancing the understanding of the psychological processes underlying employee performance in educational organizations.

## Data Availability

The raw data supporting the conclusions of this article will be made available by the authors, without undue reservation.
