# Supplementary material for: Recognition, rewards, and job performance: the mediating role of perceived organizational support in the Peruvian education sector
Source: Front Psychol. 2026 Aug 24;17:1884038. doi: 10.3389/fpsyg.2026.1884038 (PMC13547003; doi:10.3389/fpsyg.2026.1884038)
Supplement: Supplementary file 1 [file Table_1.docx]

**Apéndice A - Spanish**

**Recompensas:**

Las recompensas se distribuyen correctamente.

Las recompensas coinciden con mi esfuerzo laboral.

Estoy satisfecho con la calidad/cantidad de las recompensas.

Estoy dispuesto a aumentar mis esfuerzos de trabajo para obtener recompensas.

Los empleados trabajan más en equipo para obtener recompensas.

Las recompensas tienen un efecto positivo en el ambiente de trabajo.

Las recompensas me motivan a desempeñarme bien en mi trabajo.

**Reconocimiento:**

Soy elogiado regularmente por mi trabajo.

Recibo críticas constructivas sobre mi trabajo.

Recibo crédito por lo que hago.

Me dicen que estoy progresando.

**Apoyo organizacional percibido:**

Los beneficios que recibo en esta empresa satisfacen mis necesidades.

Esta empresa tiene una cultura que me permite desarrollar mis habilidades profesionales

Esta empresa me brinda las herramientas que necesito para ayudarme a crecer y navegar mi carrera profesional

La empresa proporciona una compensación justa y beneficios a cambio de mis contribuciones en ella.

**Rendimiento de los empleados:**

Mi desempeño es mejor que el de mis colegas con calificaciones similares.

Estoy satisfecho con mi desempeño porque en su mayoría es bueno.

Mi desempeño es mejor que el de los empleados con calificaciones similares en otras organizaciones

**Apéndice A - English**

**Rewards:**

Rewards are distributed fairly.

Rewards reflect my work effort.

I am satisfied with the quality and quantity of the rewards.

I am willing to increase my work effort to earn rewards.

Employees work more as a team to earn rewards.

Rewards have a positive effect on the work environment.

Rewards motivate me to perform well at my job.

**Recognition:**

I am regularly praised for my work.

I receive constructive feedback on my work.

I receive credit for what I do.

I am told that I am making progress.

**Perceived Organizational Support:**

The benefits I receive at this company meet my needs.

This company has a culture that allows me to develop my professional skills.

This company provides me with the tools I need to help me grow and navigate my professional career.

The company provides fair compensation and benefits in exchange for my contributions.

**Employee Performance:**

My performance is better than that of my colleagues with similar qualifications.

I am satisfied with my performance because it is mostly good.

My performance is better than that of employees with similar qualifications at other organizations.
